# Tracheal Bronchus and Associated Anomaly Prevalence Among Children

**DOI:** 10.7759/cureus.15192

**Published:** 2021-05-23

**Authors:** Amal Al-Naimi, Sara Hamad, Ahmed Abushahin

**Affiliations:** 1 Pediatric Pulmonology, Sidra Medicine, Doha, QAT; 2 Pediatric Pulmonology, Hamad Medical Corporation, Doha, QAT

**Keywords:** tracheal bronchus, trachea, airway anomalies, bronchoscopy, chromosomal abnormalities

## Abstract

Background and objectives

Tracheal bronchus (TB) is a rare congenital airway anomaly originating from the trachea, with a reported prevalence of 0.9%-3% in children. Although TB was studied in the literature, this anomaly was not evaluated in Qatar. Our study aimed to identify the prevalence and congenital anomalies associated with TB in children in Qatar.

Design

In this descriptive study, we identified patients who underwent flexible bronchoscopy (FB) at two large tertiary centers in Qatar from July 2007 to November 2020. The patients’ demographic, bronchoscopic, and radiologic data were collected. The prevalence of TB and associated congenital anomalies were determined.

Results

Of 1786 patients who underwent FB, 20 (1.12%) were diagnosed with TB. The median age at the time of diagnosis was 31 months (range, 2-154 months). The associated congenital anomalies were identified in 16 cases (80%; p = 0.007). Cardiac defects represent the most common associated anomaly (8/20, 40%).

Conclusion

This study revealed that TB is an uncommon airway anomaly and emphasizes its significant association with other congenital malformations. Our findings should alert physicians to other associated TB anomalies and provide timely management when needed.

## Introduction

Tracheal bronchus (TB) is a rare congenital tracheal anomaly, which is defined as the presence of an ectopic bronchus arising from the lateral wall of the trachea and supplies the right upper lobe [1]. In the literature, the prevalence of TB is between 0.9% and 3% [2-4]. The majority of TB cases are asymptomatic and diagnosed incidentally by advanced chest imaging or bronchoscopy [4,5].TB may present with recurrent pneumonia, cough, stridor, wheezing, bronchiectasis, or atelectasis of the affected lobe [1,6,7]. Most often, TB is associated with other congenital anomalies, such as congenital heart disease, pulmonary vasculature abnormalities, airway anomalies, or chromosomal anomalies [4,6,8].

A TB is classified as either displaced (if the right upper lobe bronchus has posterior and anterior bifurcation only) or supernumerary (if the ectopic bronchus coexists with the normal right upper lobe bronchus trifurcation); the displaced is more frequent than the supernumerary type [9]. The term pig bronchus or bronchus suis (a normal anatomy present in pigs) is often used when it supplies the entire right upper lobe (usually the right side) [1,10].

Arbitrarily, Conacher classified TB based on its origin from the trachea as follows: Type I is the TB originating at the junction of the middle and lower one-third of the trachea, type II is the TB ascending at the lower third of the trachea, and type III is the TB arising from the lower trachea, near the carina forming the carina trifurcation appearance [11].

Although TB was studied in the literature, this anomaly was not evaluated in Qatar. Therefore, our study aimed to determine the prevalence of TB and associated congenital anomalies in children who underwent flexible bronchoscopy (FB) for respiratory symptoms in Qatar. This recognition is essential to define the burden of this anomaly in our population and understand its implications.

## Materials and methods

The current study was conducted in the pediatric pulmonology unit at Hamad Medical Corporation and Sidra Medicine in Qatar. All data were collected retrospectively from our FB database, including bronchoscopies performed from July 2007 to November 2020. Children between birth and 14 years of age were included in the analysis. Patient characteristics, including age, sex, medical history, and the presence of genetic diseases, were collected along with the patients’ radiological records. Any chromosomal anomaly has already been diagnosed by karyotype or microarray by the primary care physician. Symptoms and bronchoscopy indications were recorded. 

The main outcome measures were the prevalence of TB in the study cohort and the rate of TB-related abnormalities. Statistical analysis data (continuous and categorical data) were analyzed using standard descriptive analysis. The results were expressed in absolute numbers and percentages of the whole cohort for the categorical variables and the median and interquartile ranges for the continuous variables. The categorical variables were compared with the chi-square (*x^2^*)* *test; p-values <0.05 were considered statistically significant for all analyses.

The Medical Research Center at HMC and Sidra Medicine approved the study with reference numbers MRC-01-20-1034 and 1679500, respectively. A waiver of the signed informed consent requirement was obtained as this was a retrospective descriptive study. All patient data were anonymized.

## Results

Out of 1786 FBs performed between July 2007 and November 2020, 20 TB cases were identified (1.12%). The median age of the patients at the time of diagnosis was 31 months (2-154 months); 13/20 were males (65%; p = 0.18) (Table 1). The FB indications were as follows : 1) persistent lobar collapse in seven patients, 7/20 (35%); 2) cough and wheezing in four patients, 4/20 (20%); and 3) other indications included ruling out foreign body aspiration, suspected upper airway obstruction, recurrent aspiration, and difficulty weaning from mechanical ventilation.

**Table 1:** Clinical characteristics of children with TB and its associated congenital defects TB, tracheal bronchus; TOF, tetralogy of Fallot; PDA, patent ductus arteriosus; VSD, ventricular septal defect; A-V, atrioventricular.

Variables	N or median	% or range
TB	20	1.12
Median age in months	31	2-154
Sex male	13	65
TB with associated anomalies	16	80
Chromosomal abnormalities	7	35
Trisomy 21	5	25
CHARGE association	1	5
Pierson syndrome	1	5
Cardiac defects	8	40
Cyanotic (TOF)	2	10
Non-cyanotic: PDA, VSD, A-V canal	6	30
Congenital pulmonary airway malformation	1	5
Congenital diaphragmatic hernia	1	5
Airway malformations	6	30
Laryngomalacia	1	5
Tracheomalacia	2	10
Bronchomalacia	2	10
Tracheal stenosis	1	5

**Figure 1:**
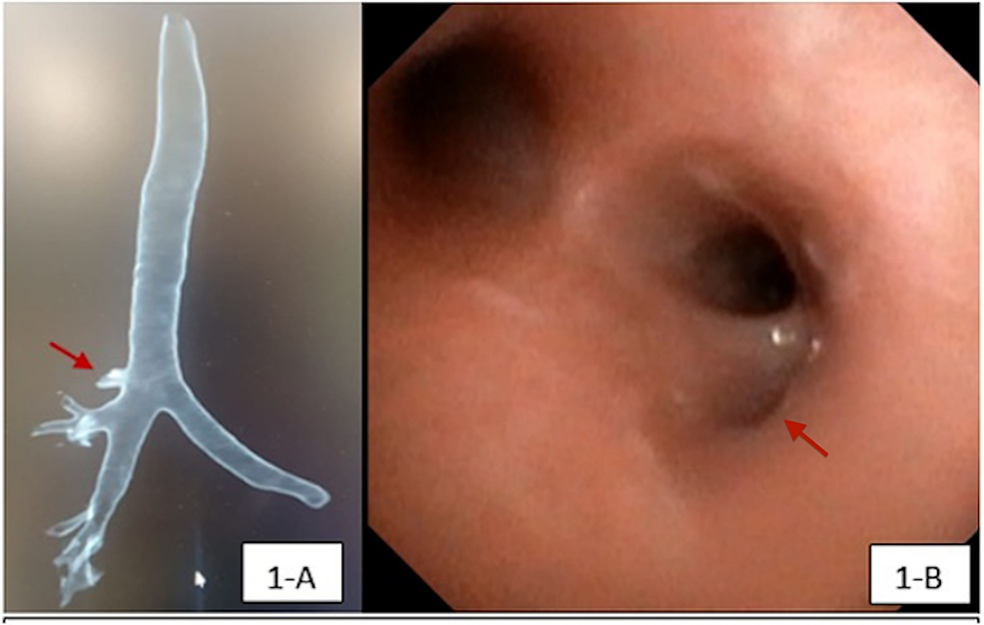
Multidetector CT chest and bronchoscopic views of a TB in a patient A. Multidetector CT scan with three-dimensional reconstruction showing right TB (red arrow);  B. Endoscopic view showing the early projection of a right TB at the lower third portion of the trachea (red arrow). TB, tracheal bronchus.

TB originated from the right side of the trachea in all patients. Most of the TB were type II, where the origin was in the lower third of the trachea. The associated congenital anomalies were observed in 16/20 patients (80%; p = 0.007); some of the patients had more than one underlying anomaly; 6/20 patients (30%) had associated airway abnormalities, mainly laryngomalacia, tracheomalacia, and bronchomalacia. Congenital syndromes were seen in 7/20 patients (35%), and cardiac defects were detected in 8/20 patients (40%). Trisomy 21 was the most prevalent condition (n = 5/20, 25%). Table 1 shows the demographics of the cohort and distribution of TB-associated congenital anomalies. Eight out of the 20 patients (40%) had a conventional CT chest before FB, and in 6/8 patients (75%), TB was noted in the CT images and confirmed by FB, as illustrated in Figure 1A, B. No surgical intervention was required in any case.

## Discussion

TB is a rare congenital lower airway malformation, with a prevalence of 0.9%-3% worldwide [2-4]. TB is defined as an aberrant bronchus that originates from the lateral wall of the trachea above the carina and supplies the upper lung lobe [1,9]. In our study, the prevalence of TB was 1.12%, which falls within the reported range in the literature. Right TB is more frequent than the left TB with a reported prevalence of 0.1%-2% and 0.3%-1%, respectively [2,12]. We detected only right-sided TB arising from the lower third of the trachea in our study.

The exact pathogenesis of TB remains unclear, but it is secondary to defects occurring in utero between the third and 16th weeks of gestation, and it is often accompanied by other congenital malformations [12], most commonly laryngomalacia, tracheomalacia, tracheal stenosis, congenital heart disease, and genetic disorders, particularly trisomy 21 [3,4,13]. In our cohort, 16 out of 20 cases of TB (80%; p = 0.007) had associated congenital abnormalities, such as congenital heart disease (40%), chromosomal anomalies (35%), and airway anomalies (30%). Other abnormalities such as CHARGE association (5%) and congenital diaphragmatic hernia (5%) were also found. Tracheal bronchi are often asymptomatic and discovered incidentally during bronchoscopy or radiological workup performed for various respiratory issues [4,5]. When symptomatic, TB may cause recurrent pneumonia or atelectasis, stridor, wheezing, and persistent cough [1,6,7]. Due to its retrospective nature and the small sample size, it is difficult to conclude if the TB in our patients was the actual cause of the respiratory symptoms or just a coincidental finding. However, the link between TB and respiratory symptoms has been proposed because TB is accompanied by altered airway geometry and trapped mucus [6,7].

In the setting of TB, it is worth mentioning that endotracheal intubation merits special consideration. Misplacement of the endotracheal tube can occlude the TB lumen, resulting in atelectasis, hypoxia, or even respiratory insufficiency [14,15]. Hence, knowledge of TB anatomy is essential perioperatively to take the necessary precautionary measures and avoid complications [2,16].The diagnosis of TB can be achieved through high-resolution CT scans of the chest or by bronchoscopy [5,7]. In our study, the chest CT scan was able to detect the TB anomaly in six out of eight confirmed cases. This finding supports the use of non-invasive imaging as a first-line tool to diagnose TB when clinically suspected. Imaging can also evaluate the lung parenchyma to rule out any possible complications, such as bronchiectasis, focal emphysema, and focal cystic lesions [17]. Novel multidetector CT with 3D image reconstruction can define the bronchus origin and location, thus limiting the need for FB [5,17,18].

TB is asymptomatic in the majority of the cases and does not require intervention [7]. However, surgical excision of TB along with the associated lobe is reserved for selected cases of recurrent pneumonia, bronchiectasis, or chronic respiratory symptoms [19]. In our cohort, no surgical intervention was required in any case, indicating a low disease burden on the healthcare system in our community.

## Conclusions

This study revealed that TB is an uncommon airway anomaly and emphasizes its significant association with other congenital malformations. Therefore, even though this anomaly appears to carry a low disease burden in Qatar, our findings should alert physicians to other associated TB anomalies and provide timely management when needed.
